# The Joint Effect of Paternal and Maternal Parenting Behaviors on School Engagement Among Chinese Adolescents: The Mediating Role of Mastery Goal

**DOI:** 10.3389/fpsyg.2019.01587

**Published:** 2019-07-10

**Authors:** Juan Wang, Xinxin Shi, Ying Yang, Hong Zou, Wenjuan Zhang, Qunxia Xu

**Affiliations:** ^1^Institute of Developmental Psychology, Beijing Normal University, Beijing, China; ^2^Beijing Key Laboratory of Applied Experimental Psychology, Faculty of Psychology, Beijing Normal University, Beijing, China; ^3^School of Behavioural and Health Sciences, Australian Catholic University, Sydney, NSW, Australia; ^4^Department of Psychology, School of Philosophy, Wuhan University, Wuhan, China

**Keywords:** paternal parenting behaviors, maternal parenting behaviors, school engagement, mastery goal, adolescents

## Abstract

The present study aimed to explore the joint effect of paternal and maternal parenting behaviors on adolescent’s school engagement, and the mediating role of mastery goal. A total of 2,775 Chinese adolescent participants (55.3% females, mean age = 15.70, *SD* = 1.57) from two-parent families were recruited in 2014, who rated their perceptions of emotional warmth, behavioral guidance, harsh discipline of their father and mother, as well as their own mastery goal and school engagement. Results showed that paternal and maternal parenting behaviors had interaction effects on school engagement with different interaction patterns. Specifically, the interactions of both parents’ emotional warmth and both parents’ behavioral guidance displayed strengthening patterns, where one parent’s high emotional warmth or behavioral guidance enhanced the positive relationship between the corresponding parenting behavior of the other parent and adolescents’ school engagement. By contrast, the interaction of both parents’ harsh discipline displayed an interfering pattern, where one parent’s high level of harsh discipline reduced the negative relationship between harsh discipline of the other parent and school engagement. Further, all three interaction effects between father and mother on school engagement were mediated by mastery goal. These findings underline the importance of viewing family from a systematic perspective and the benefits of supportive parenting behavior of both parents.

## Introduction

School engagement is a vital and positive index of students’ school lives (Schaufeli et al., 2002) and is defined as the quality of students’ involvement with the endeavor of schooling, including cognitive, affective, and behavioral engagement (Fredricks et al., 2004). Prior studies have revealed that students who are more actively engaged in school achieve higher grades, show better school adjustment, and tend to become competent members of the society (Wang and Holcombe, 2010; Li and Lerner, 2011). Meanwhile, school engagement is found to be negatively related to ages (Wang and Eccles, 2012) and exist significant individual differences (Janosz et al., 2008) in adolescence. Thus, it’s necessary to examine the factor influencing adolescents’ school engagement.

Parenting behaviors refer to the specific, goal-directed behaviors that parents use to socialize their children (Prevatt, 2003), and are identified as a vital family context that can influence adolescents’ school engagement. Inspired by family system theory (Bornstein and Sawyer, 2005), which suggests that a father and a mother make a joint contribution to their offspring’s academic development, previous studies have found that the relationship between one parent’s parenting behavior and developmental outcomes can be moderated by the parenting behavior of the other parent (McKee et al., 2007; Lowe and Dotterer, 2013; Foster et al., 2016). However, these studies focused on offspring’s socioemotional functioning or academic outcomes, it is unclear how paternal and maternal parenting behaviors have a joint of effect on adolescents’ school engagement. To address this gap, this study aims to investigate whether there exists the interaction effect between the similar type of paternal and maternal parenting behaviors on adolescents’ school engagement. If there exists, which mediator can make it work? Mastery goal, referring to the motivation to develop competence, is shown to play a mediating role in the relationship between parenting behaviors and school engagement (Skinner et al., 2009; Luo et al., 2013). In order to reveal the motivational mechanism, the present study also wants to examine the role of mastery goal on how the interaction effects between paternal and maternal parenting behaviors on school engagement.

### Parenting Behaviors and School Engagement

Parents are the first teachers of their children’s, their parenting behaviors have a profound influence on individual achievement-related outcomes (Spera, 2005; Castro et al., 2015; Vasquez et al., 2016; Garrett-Peters et al., 2019). Different parenting behaviors may have different effects on adolescents’ school engagement. Some parenting behaviors are supportive, such as warmth or autonomy support (e.g., providing warmth, love, care, and encouragement of autonomous behaviors), which can improve offspring’s academic development (Joussemet et al., 2008; Hill and Wang, 2015; Doctoroff and Arnold, 2017). However, some parenting behaviors such as physical punishment (e.g., spanking, hitting) and psychological control (e.g., guilt induction, love withdrawal, shaming) are considered as non-supportive and detrimental to offspring’s school performance (Joussemet et al., 2008; Su et al., 2015; Wang et al., 2018). Physical punishment and psychological control are similar in essence, as both can control the child through parental authority and may cause resentment or aversive (Nelson et al., 2006). The two forms of punishment can be integrated as harsh discipline (Wang and Liu, 2014), which has proved to be a typical manifestation of Chinese parenting culture (Wang and Liu, 2014; Wang et al., 2017). Specifically, it is defined as that parents impose their own will on their children with non-supportive strategies such as punishment or withdrawal care to control behaviors of their children.

Different parenting cultures have different views on behavioral control, which causes the controversial effect of behavioral control (Chao, 2001; Gershoff et al., 2010; Helwig et al., 2014). Behavioral control in Western culture is defined as parental behaviors that attempt to control or manage children’s behavior by rules and restrictions (Barber, 1996). One example is “My parents asked me where I went with my friends.” According to Western definition, monitoring and rule setting is emphasized in the content of behavioral control (Pomerantz and Wang, 2009), and prior studies have shown that this concept is unrelated to academic achievement (e.g., Bean et al., 2003, 2006), and may even have a slight negative association with academic achievement (Kramer, 2012). However, in Chinese culture, parents are considered to be responsible for teaching their offspring so that the young and dependent child can become a qualified economic and social success (Wu, 1996). It is also believed that behavioral control over children’s activities and behaviors in the physical world provides children with needed guidance (Wang et al., 2007), and is also seen as a predictive variable for better academic achievement (Chao, 1994; Lee et al., 2012). In this study, the term behavioral guidance is used instead of behavioral control, which emphasizes the culture of teaching or training. It means that parents train children’s sense of rules and behavioral habits to conform to social norms.

Although China has the largest population in the world (Sangawi et al., 2015), most research on parents’ role in children’s behavior and achievement outcomes are based on Western parenting culture (Kim and Wong, 2002; Hill and Wang, 2015, pp. 185). Thus, the present study aims to investigate whether parenting behaviors (emotional warmth, behavioral guidance, harsh discipline) have an effect on school engagement in the Chinese cultural context. We hypothesize that emotional warmth and behavioral guidance are positively associated with school engagement, but harsh discipline is negatively associated with school engagement (hypothesis 1 or H1).

### The Joint Effect of Father and Mother

As the involvement of fathers in parenting becomes more popular (Sarkadi et al., 2008; Martin et al., 2010; Jeynes, 2015), it is important to consider the joint contribution of both parents to their offspring. According to Bronfenbrenner’s (1986) ecological system theory, both father and mother are important microsystem partners for children’s development, which underlines the importance of combining the effect of paternal and maternal parenting behaviors (Pleck, 2007, a review). Family system theory further declares that the family system is an organized whole, and its subsystems, including individuals and their relationships, are interdependent and dynamic. Based on this, some scholars investigated the joint contribution of paternal and maternal parenting to their offspring’s psychosocial adjustment by analyzing their interaction with each other (Li and Meier, 2017, a review; Papadaki and Giovazolias, 2015); other studies supported the interaction effect on school performance (Lowe and Dotterer, 2013;Babinski et al., 2017).

The interaction of the parenting behaviors of fathers and mothers may follow three patterns. The first is *strengthening pattern*, which means that the association between academic development and one supportive parent can be intensified when the other parent is also supportive. The second pattern is *buffering pattern*, which occurs when one parent is non-supportive, the other parent’s supportive behavior then plays a protective role in offspring’s academic development. The third pattern is *interfering pattern*, that is, the influence of one parent on academic development may be hindered or reduced by the other parent. This pattern is relatively rare but possible.

Although there are some valuable studies on the interactions of maternal and paternal parenting, these studies focused on offspring’s socioemotional functioning or academic outcomes (McKee et al., 2007; Lowe and Dotterer, 2013; Foster et al., 2016). There is insufficient research on whether paternal and maternal parenting behaviors will also have an interaction effect on adolescents’ school engagement. Therefore, the current study aims to address this gap by testing whether the effect of one parent’s parenting behavior on adolescent’s school engagement is moderated by the corresponding parenting behavior of the other parent (e.g., father’s emotional warmth, and mother’s emotional warmth). Given that strengthening, buffering, and interfering patterns of interactions are all plausible, we assume that the interactions between father and mother are significant (hypothesis 2 or H2), but do not assume that the interactions will take on a specific pattern.

### Motivational Mechanism of Mastery Goal

Although the joint effect of paternal and maternal parenting behaviors may plausibly explain the differences in adolescents’ school engagement, it remains unclear how adolescents’ motivational factor may affect the relationship between two parents’ parenting behaviors and school engagement. The self-system model of motivational development posits that motivational factors such as goal orientation can contribute to the quality of individual engagement (Deci and Ryan, 2000; Skinner et al., 2009). Many researchers support a linear and temporal order of engagement-related processes which can be described as context → motivation → engagement. That is, students’ motivation can be shaped by the quality of the context they interact with, which then influences their engagement in learning and subsequent development outcomes (Skinner et al., 2009; Lawson and Lawson, 2013). Under this framework, the mediating role of mastery goal in the relationship between family context and academic performance has gradually become a research hotspot (Skinner et al., 2009; Luo et al., 2013;Chen, 2015).

Mastery goal represents students’ motivation to develop competence (Ames and Archer, 1988), which is proven to be associated with adaptive patterns of learning by experimental, correlational, as well as qualitative research (Kaplan and Maehr, 2007, a review). Mastery-oriented students tend to spend more time studying with their own initiative, persist longer in the face of difficulties, report greater interest and effort, employ deep learning strategies more frequently (Liem et al., 2008; Benita et al., 2014). Therefore, mastery goal is identified as a beneficial goal approach to improve students’ school engagement, the higher level of mastery goal, the more actively engaged in learning tasks (Gonida et al., 2007, 2009; Kaplan and Maehr, 2007).

The development of mastery goal during the school years may be explained by parenting behaviors. Parental involvement, autonomy support, and warmth can prompt the formation of mastery goal, whereas punishment and psychological control are found to be non-significant associated with mastery goal (Duchesne and Ratelle, 2010; Fletcher et al., 2012; Chen, 2015; Diaconu-Gherasim and Mãirean, 2016). However, the role of parental behavioral control in shaping mastery goal is ambiguous. For instance, a study conducted in the Chinese sample found that authoritarian, a kind of parenting style characterized by punishment and strict enforcement, is unrelated to mastery goal (Chen, 2015). Another study focuses on parental coercive discipline also found similar results in the Singapore sample (Luo et al., 2013). By contrast, a study within the Australian context found that parental monitoring is positively associated with mastery goal (Boon, 2007). Luo et al. (2013) further indicated that parental involvement could affect children’s mastery goal, and mastery goal could, in turn, promote children’s engagement in classwork and homework, but mastery goal could not mediate the relationship between parental coercive discipline and engagement.

In short, different parenting behaviors have different roles in shaping mastery goal, and then produce an impact on academic behavior and outcomes. Based on this premise, this study also aims to explore the mediating role of mastery goal. Referring to the results of Luo et al. (2013), mastery goal is supposed to play a mediator in the relationship between parental emotional warmth and school engagement, but not in the relationship between parental harsh discipline and school engagement in the present study. And due to behavioral guidance was defined as positive parenting in this study, it is assumed to be positively linked with mastery goal, and then foster school engagement (hypothesis 3 or H3). In addition, as aforementioned, paternal and maternal parenting behaviors may have an interaction effect on school engagement, whether the interaction effect will be mediated by mastery goal is unclear. To address this issue, mediated moderation models will be tested in this study. We hypothesize that the interaction effect of paternal and maternal parenting behaviors on mastery goal will be significant, but the residual interaction effect on school engagement will be reduced or non-significant (hypothesis 4 or H4).

### The Present Study

To understand whether and how paternal and maternal parenting behaviors have a joint effect on adolescents’ school engagement, we aim to explore the interaction effect between paternal and maternal parenting behaviors on adolescents’ school engagement based on family system theory. Further, based on the self-system model of motivational development, we also hope to examine the mediating effect of mastery goal on the link between paternal parenting behavior, maternal parenting behavior, the interaction term of father and mother, and school engagement. Based on this, we proposed the following four hypotheses:

Hypothesis (*H1*). Parental emotional warmth and behavioral guidance will positively predict school engagement; harsh discipline will negatively predict school engagement, regardless of the sex of the parents.
Hypothesis (*H2*). There will be significant interaction effects between paternal and maternal parenting behavior on adolescents’ school engagement. However, the interaction effect of different pairs of parenting dimensions will display different patterns.
Hypothesis (*H3*). Mastery goal will mediate the relationship between emotional warmth, behavioral guidance and school engagement, but will not mediate the relationship between harsh discipline and school engagement.
Hypothesis (*H4*). Mastery goal will also mediate the relationship between the interaction terms of each pair of paternal and maternal parenting dimension and school engagement.

## Materials and Methods

### Participants

Participants were middle and high school students from a broader project focusing on the relationship between family environment and students’ mental health. In total, 3,080 adolescents participated in this study. They were from eight public middle/high schools (108 classes), covering three urban districts and three rural districts of Beijing, China. Because the goal was to explore the interaction between paternal and maternal parenting, data of participants from one-parent families were excluded. In sum, data of 2,775 participants from two-parent families were adopted in this study. Their ages ranged from 10.75 years old to 18.92 years old. Participants were from four grades, including grade 7 (*N* = 521, *M*_age_ = 13.43 years, *SD* = 0.48), grade 8 (*N* = 553, *M*_age_ = 14.36 years, *SD* = 0.47), grade 10 (*N* = 941, *M*_age_ = 16.40 years, *SD* = 0.45), and grade 11 (*N* = 760, *M*_age_ = 17.35 years, *SD* = 0.47). Because of imminent graduation, students from grade 9 and grade 12 grades were not included in this survey. Although 34 of 2,775 participants were not able to complete the study questionnaires due to conflicting tasks or emergencies, the missing data represented only a small percentage (1.2%) of the data and was handled with full information maximum likelihood (FIML) procedures. FIML is a model-based parameter estimation method, of which estimates are computed by maximizing the likelihood of a missing value based on observed values in the data. It has been suggested that FIML approach can maximize the use of available data information and produce unbiased estimates under ignorable missing data conditions (Enders and Bandalos, 2001).

### Procedure

This study was approved by the Ethics Committee of the Faculty of Psychology, Beijing Normal University. Because the potential risk of the protocol was low and the data collection was anonymous, the letter that described the study and consent forms were only sent to school administrators and teachers. Before the data collection, the class adviser sent a message to tell parents about the purpose and voluntary nature of this survey in the Parents WeChat Group. All parents responded in the WeChat Group that they had been informed and agreed to their children’s participation in this survey. Students were also informed of the purpose and voluntary nature of the survey and their right to withdraw at any time. All voluntary participants completed a self-reported questionnaire booklet in the quiet of their classrooms. The questionnaires were administered by the first author and postgraduate students in Psychology who received training. It took approximately 20 min for students to complete the survey. Students received small gifts for their participation.

### Measures

#### Parenting Behaviors

Due to the difference in parenting culture, a parenting behavior scale adapted to the characteristics of Chinese parenting culture is needed. Based on the existing constructs and content of classical parenting style scales such as the Egma Minnen av Bardndosnauppforstran (EMBU, Perris et al., 1980) and the Ghent Parental Behavior Scale (GPBS, Van Leeuwen, 1999), a new and more concise parenting behavior scale was developed in this study. The new scale includes three dimensions, emotional warmth, behavior guidance, and harsh discipline.

In China, parents emphasize the importance of loving and caring child as parents in European American (Chao, 1995; Chao and Tseng, 2002). They spend time with their children, encourage children’s autonomous behaviors, which is similar to the measure content of emotional warmth dimension of EMBU and positive parenting of GPBS. We integrated their contents and used the name of emotional warmth in the present study. Behavioral guidance, a new dimension in this study, refers to parental guidance and training in children’s sense of rules and behaviors habits. Different from the typical measurement of behavioral control in Western culture which emphasizes monitoring and rule setting (Barber, 1996), the content of behavioral guidance reflects teaching or guidance in Chinese parenting culture. Items of behavioral guidance were adapted and developed from Behavioral Control Scale (Wang et al., 2007) and the rules dimension of GPBS (Van Leeuwen, 1999). Harsh discipline is an integrated concept of physical punishment and psychological control, which means parents impose their own will on their children with non-supportive strategies. Items of harsh discipline were adapted from negative control factor of GPBS (Van Leeuwen and Vermulst, 2004) and Psychological Control Scale (Wang et al., 2007).

The final scale includes 21 items, seven items for emotional warmth (e.g., “My father/mother does activities together with me, because they know that I enjoy it, such as sports, walking, shopping”), five items for behavior guidance (e.g., “father/mother teaches me to be polite to others”), and nine items for harsh discipline (e.g., “My father/mother often blame me for being lazy and useless in front of others”). Participants were asked to evaluate their paternal and maternal parenting behavior separately, and rated each item on a five-point Likert scale ranging from very strongly disagree (1) to very strongly agree (5).

Prior to the formal study, we collected responses from 556 adolescents to test the construct validity of the scale. Exploratory factor analysis (EFA) showed that the three factors model of both father (*χ*^2^(150) = 279.43, *p* < 0.001; RMSEA = 0.04; CFI = 0.96; TLI = 0.95) and mother (*χ*^2^(150) = 284.240, *p* < 0.001; RMSEA = 0.04; CFI = 0.96; TLI = 0.95) were supported, and factor loadings varied from 0.376 to 0.818 for all items. In the formal study, the new scale was also proved to have good validity and reliability. The results of confirmatory factor analysis (CFA) for both parents were acceptable (father: *χ*^2^(186) = 1072.35, *p* < 0.001; RMSEA = 0.04; CFI = 0.95; TLI = 0.95; mother: *χ*^2^(186) = 1000.483, *p* < 0.001; RMSEA = 0.04; CFI = 0.95; TLI = 0.95). Cronbach alpha coefficients varied from 0.83 to 0.88.

#### Mastery Goal

The Achievement Goal Orientation scale developed by Elliot and Thrash (2002) was proven to be applicable to Chinese culture (Lau and Lee, 2008). The mastery goal dimension of this scale was used in this study, including 5 items (e.g., “I like to learn something really challenging in class so that I can learn something new”). Participants were asked to indicate their agreement on a five-point Likert type scale, ranging from unlike me (1) to very much like me (5). The Cronbach alpha coefficient of mastery goal was 0.78.

#### School Engagement

The Student Engagement Questionnaire developed by Lam et al. (2012) and revised by Chinese researchers (Ma et al., 2015) was used to measure school engagement. The questionnaire consisted of 16 items across three dimensions: behavioral engagement (e.g., “I try hard to do well in school”), cognitive engagement (e.g., “When I study, I try to connect what I am learning with my own experiences”), and affective engagement (e.g., “I like what I am learning in school”). Participants were asked to indicate their agreement on a five-point Likert type scale, varying from unlike me (1) to very much like me (5). This scale demonstrated good internal reliability, as Cronbach alpha coefficients of three dimensions varied from 0.88 to 0.91. The Cronbach alpha of the full scale was 0.93.

#### Covariates

Gender, age, and socio-economic status (SES) were considered as covariates to partial out their possible impacts on parenting behavior, mastery goal, and school engagement (Hoff et al., 2002; Walker et al., 2006; Pellerone et al., 2018). SES information was reported by students, including their parental education level and occupations, respectively and the monthly income of both father and mother.

Prior to formal data analysis, indicators of SES were assigned (Shi and Shen, 2007). Education level was coded from 1 to 4 (1 = junior middle school education or below, 2 = high school or technical school education, 3 = Bachelor’s degree, 4 = Master’s degree or above); occupations were coded from 1 to 5 (1 = unemployed or temporary work, 2 = manufacturing or service, 3 = office work, 4 = administrative or managerial, 5 = professional and technical); and family monthly income was coded from 1 to 7 (1 = relying on government relief, 2 = less than 3,000 RMB, 3 = 3,000 to 5,000 RMB, 4 = 5,000 to 8,000 RMB, 5 = 8,000 to 12,000 RMB, 6 = 12,000 to 20,000 RMB, 7 = more than 20,000 RMB). The number and ratio of each category of SES characteristics can be seen in Table 1. After coding, the five indicators of SES were standardized separately, and the principal component analysis was applied to obtain factor loadings of each indicator. Finally, the total family SES was synthesized with factor loadings as the weight (Bradley and Corwyn, 2002).

**Table 1:** Socio-economic status characteristics of participants’ parents.

		Mother		Father		Family	
		*n*	%	*n*	%	*n*	%
**Educational level**							
≤junior middle school		926	33.37	874	31.50		
high or technical school		985	35.50	993	35.78		
Bachelor’s degree		740	26.67	697	25.12		
≥Master’s degree		124	4.47	211	7.60		
**Occupation**							
unemployed or temporary work		416	14.99	145	5.23		
manufacturing or service		663	23.89	823	29.66		
office work		951	34.27	970	34.95		
administrative or managerial		447	16.11	411	14.81		
professional and technical		298	10.74	427	15.39		
**Monthly income**							
relying on government relief						22	0.79
<3,000 RMB						267	9.62
3,000–5,000 RMB						731	26.34
5,000–8,000 RMB						789	28.43
8,000–12,000 RMB						476	17.15
12,000–20,000 RMB						261	9.41
>20,000 RMB						228	8.22

### Analytic Plan

To avoid the potential for a common method bias caused by self-report, we adopted an anonymous measurement and conducted Harman’s single-factor test. All items in this study were loaded into an EFA and the results revealed the presence of ten factors with initial eigenvalues greater than 1.00. The first factor accounted for 21.69% of the variance, suggesting that the influence of common method variance was quite small (Podsakoff et al., 2003).

Three steps were used to investigate whether and how paternal and maternal parenting behaviors interacted with each other as they impact adolescents’ school engagement, and whether the interaction effects on school engagement will be mediated by mastery goal. First, descriptive statistics were presented to help understand the subsequent results. Second, to examine whether paternal and maternal parenting behaviors have a unique effect (H1) and an interaction effect (H2) on school engagement, a simple moderation model with only one dimension of paternal and maternal parenting behavior, their interaction term (the product of two predictors), school engagement, and control variables (age, gender, SES) were established. When the interaction effect was significant, the Johnson-Neyman technique was used to probe when (at what point) the relationship between paternal parenting behavior and school engagement was changed by maternal parenting behavior (Preacher et al., 2007). Third, mastery goal was integrated into simple moderation model to examine its mediating effect on school engagement. In fact, the current model is a mediated moderation model. We tested whether each pair of parental and maternal parenting dimension and their interaction term have an indirect effect on school engagement via mastery goal (H3 and H4).

Mplus 8 was adopted in this study. Because subjects were clustered into classrooms, TYPE = COMPLEX and CLUSTER = Class were set. In addition, Robust maximum likelihood estimation (MLR) was used to produce *χ*^2^ test statistics for data with non-normal and non-independence of observations (Benner et al., 2008). All variables, except for control variables, were latent structural, and the latent interaction term was estimated with the XWITH command, using FIML estimation with robust standard errors. In addition, due to Mplus software cannot provide fitting indices required to assess the validity of model with the latent interaction term, the model fitting was assessed by referring to the method proposed by Maslowsky et al. (2015). Specifically, ensuring there are qualified fitting indices of the model without latent interaction term at first. Next, the value of *D* was computed by comparing the log-likelihood values of two models with (M1) and without (M0) latent interaction term. *D* = -2 [(log-likelihood for M0) – (log-likelihood for M1)]. According to Maslowsky et al. (2015), the values of *D* can be compared to a Chi-Square distribution using *df* = 1. If the log-likelihood ratio test is significant, indicating the model with the latent interaction term is a well-fitted model.

## Results

### Descriptive Statistics

A paired samples *t*-test was conducted to compare paternal and maternal parenting behaviors. As predicted, compared to fathers, mothers were perceived to provide higher emotional warmth [*t* = -24.92, *p* < 0.001, 95% CI: (-0.33, -0.28)], behavioral guidance [*t* = -10.17, *p* < -0.001, 95% CI: (-0.11, -0.08)], as well as harsh discipline [*t* = -7.47, *p* < 0.001, 95% CI: (-0.11, -0.06)]. The means, standard deviations, and inter-correlations for all variables were presented in Table 2.

**Table 2:** Inter-correlations of the variables.

	*M* (*SD)*	1	2	3	4	5	6	7	8	9	10
(1) F-warmth	3.52 (0.77)										
(2) M-warmth	3.69 (0.71)	0.75***									
(3) F-guidance	3.85 (0.70)	0.66***	0.53***								
(4) M-guidance	4.03 (0.63)	0.54***	0.66***	0.79***							
(5) F-harsh	2.38 (0.76)	–0.47***	–0.37***	–0.29***	–0.25***						
(6) M-harsh	2.44 (0.75)	–0.37***	–0.48***	–0.22***	–0.26***	0.75***					
(7) mastery goal	3.72 (0.76)	0.27***	0.26***	0.24***	0.26***	–0.14***	–0.15***				
(8) school engagement	3.61 (0.79)	0.34***	0.33***	0.30***	0.30***	–0.21***	–0.22***	0.67***			
(9) age	15.67 (1.58)	0.02	0.05**	0.00	0.00	–0.05**	–0.07***	–0.07***	–0.08***		
(10) gender		0.07***	0.09***	0.35	0.56**	–0.18***	–0.10***	–0.03	0.01	0.01	
(11) SES		0.15***	0.14***	0.13***	0.13***	–0.10***	–0.10***	0.05***	0.14***	0.23***	–0.01

*^∗∗^*p* < 0.01, ^∗∗∗^*p* < 0.001.*

### Simple Moderation Model

In order to test whether paternal and maternal parenting behaviors have a unique effect (H1) and an interaction effect (H2) on school engagement, three simple moderation models were examined. In each model, school engagement was the outcome variable, and a pair of paternal and maternal parenting dimension, as well as their latent interaction term were the predictive variables. Before testing the hypotheses, the log-likelihood ratio test demonstrated that all three models with latent interaction term were well-fitted (Table 3). Results of path analysis supported both H1 and H2. In the model of emotional warmth, after controlling for age, gender and SES, paternal emotional warmth still positively predicted school engagement (β = 0.16, *p* < 0.001), as did maternal emotional warmth (β = 0.25, *p* < 0.001). The interaction effect between paternal and maternal emotional warmth was also significant (β = 0.07, *p* = 0.002). The model of behavioral guidance had similar results, where both paternal and maternal behavioral guidance positively predicted school engagement (father: β = 0.19, *p* < 0.001; mother: β = 0.26, *p* < 0.001), as well as their latent interaction term (β = 0.08, *p* < 0.001). In the model of harsh discipline, the main effect of the mother on school engagement was significant (β = -0.17, *p* = 0.001), and that of the father was non-significant (β = -0.06, *p* = 0.213). The interaction effect between paternal and maternal behavioral guidance on school engagement was significant (β = 0.08, *p* = 0.004).

**Table 3:** Model fit indices of simple moderation models and mediated moderation models.

			*χ*^2^/*df*	RMSEA	CFI	TLI	SRMR	Log-likelihood	*D*
Simple moderation models	emotional warmth	M0	7.10	0.05	0.96	0.95	0.03	–51130.26	14.73
		M1						–51122.89	
	behavioral guidance	M0	3.38	0.03	0.99	0.98	0.02	–38459.41	19.34
		M1						–38449.74	
	harsh discipline	M0	3.69	0.03	0.97	0.97	0.03	–65310.86	15.93
		M1						–65302.90	
Mediated moderation models	emotional warmth	M0	7.82	0.05	0.94	0.93	0.04	–68289.04	25.48
		M1						–68276.30	
	behavioral guidance	M0	6.62	0.05	0.95	0.94	0.03	–55611.48	26.04
		M1						–55598.46	
	harsh discipline	M0	5.05	0.04	0.95	0.94	0.04	–82478.26	15.83
		M1						–82470.35	

*In this table, Mo and M1 represent the model without and with the latent interaction term, respectively. *D* = -2 [(log-likelihood for M0) – (log-likelihood for M1)]. In this study, all the log-likelihood ratio tests were significant, suggesting models with latent interaction term are well-fitted.*

**FIGURE 1:**
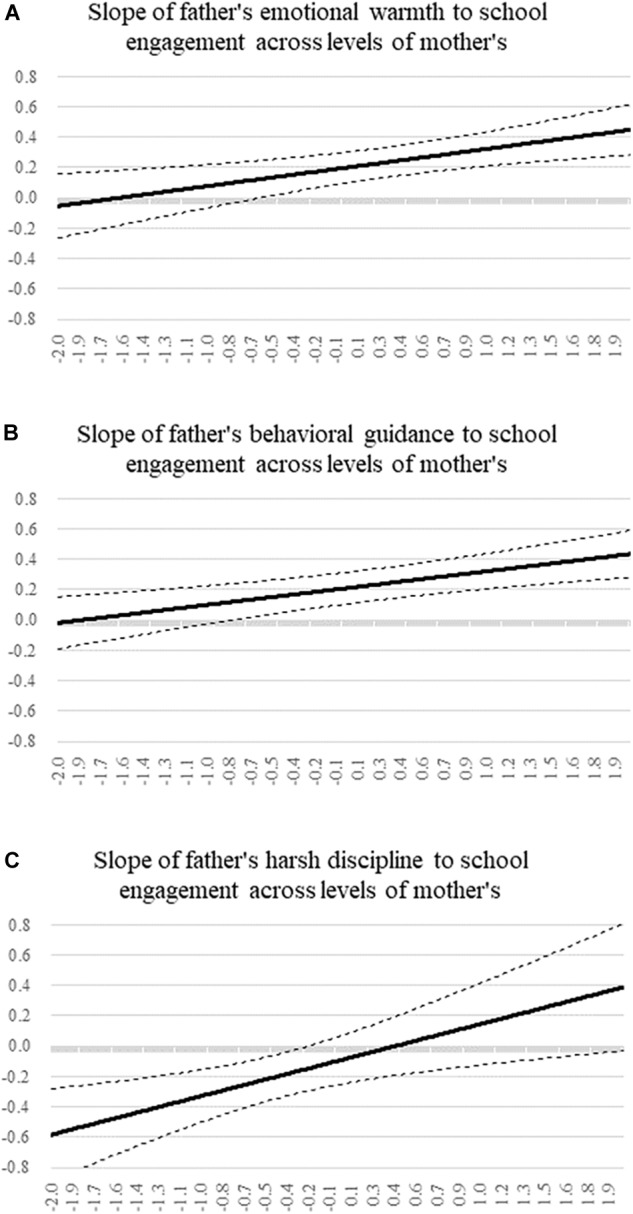
The simple slope of father’s parenting dimensions to school engagement across levels of mother’s in simple moderation models. **(A)** The slope of father’s emotional warmth to school engagement across mother’s scores. **(B)** The slope of father’s behavioral guidance to school engagement across mother’s scores. **(C)** The slope of father’s harsh discipline to school engagement across mother’s scores.

Figure 1 shows the interpretation of the interactions by the Johnson-Neyman technique to plot changes in the association between paternal parenting dimension and school engagement according to the level of the corresponding maternal dimension. The y-axis represents the standardized slope for paternal parenting dimension, and the x-axis represents data within 2 standard deviations of the mean of the corresponding maternal dimension. The solid lines represent the simple slope estimates for paternal parenting dimension, and the dotted lines represent the 95% CI around the estimates. Based on the plot of Figure 1A, the positive association between paternal emotional warmth and school engagement increased as maternal emotional warmth improved, which was in accordance with the strengthening pattern. The simple slope of paternal emotional warmth was positive and significantly different from zero when maternal emotional warmth was equal to or over -0.58 units. A similar pattern was seen in Figure 1B, that is, the predictive effect of paternal behavioral guidance on school engagement increased as maternal behavioral guidance increased, and the turning point was -0.77 units. By contrast, Figure 1C showed that the negative association between paternal harsh discipline and school engagement decreased when maternal harsh discipline increased, which was consistent with the interfering pattern. The simple slope of paternal harsh discipline was non-significant when a mother’s score was over -0.21 units.

### Mediated Moderation Model

To test whether the moderating effect of maternal parenting dimension would be mediated by mastery goal, three mediated moderation models were examined. Mediated moderation was indicated if the estimation results met three criteria: (1) the latent interaction term of paternal and maternal parenting dimension significantly predicted mastery goal; (2) the mastery goal significantly predicted school engagement; (3) the predictive effect of latent interaction term on school engagement declined in magnitude (or rendered non-significant) in comparison with the same coefficient estimated in the simple moderation model (Muller et al., 2005).

**FIGURE 2:**
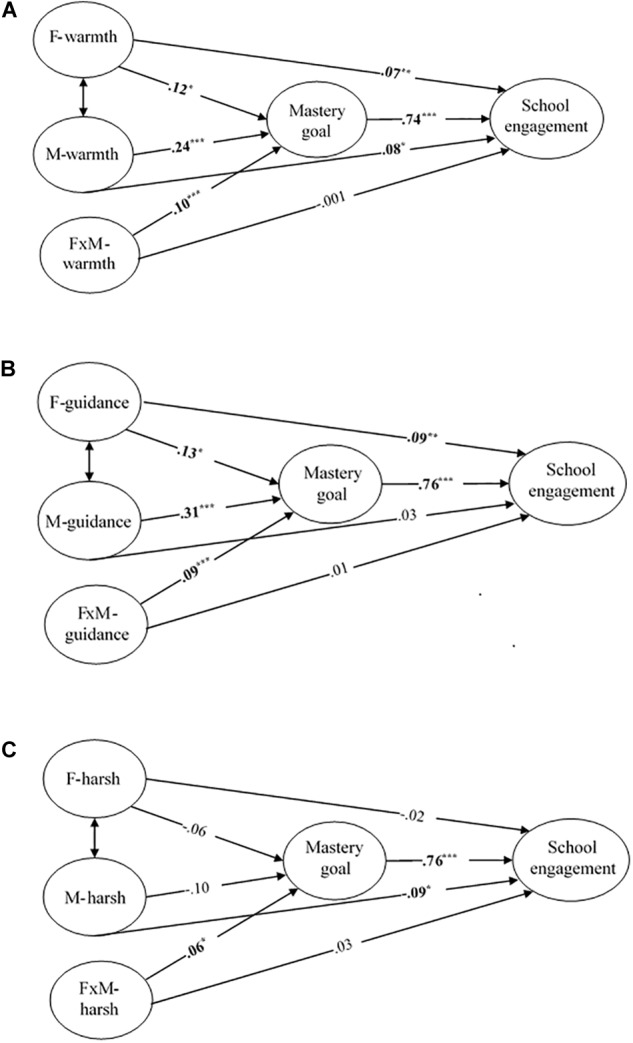
Mediated moderation models. **(A)** Mediated moderation model of paternal and maternal emotional warmth. **(B)** Mediated moderation model of paternal and maternal behavioral guidance. **(C)** Mediated moderation model of paternal and maternal harsh discipline. Bold digits indicate significant path coefficients (^∗^*p* < 0.05, ^∗∗^*p* < 0.01, ^∗∗∗^*p* < 0.001). For simplicity, all path coefficients of covariates and correlation of residuals are not presented.

As expected, the log-likelihood ratio test demonstrated that all three mediated moderation models presented qualified model fit (Table 2) and the model structures could be seen in Figure 2. Results found evidence for significant interactions between each pair of paternal and maternal parenting dimension for mastery goal (emotional warmth: β = 0.10, *p* < 0.001; behavioral guidance: β = 0.09, *p* < 0.001; harsh discipline: β = 0.06, *p* = 0.047), which indicated that the first criterion was met. In addition to the interaction effect, the main effects of both paternal and maternal emotional warmth on mastery goal were significant (father: β = 0.12, *p* = 0.022; mother: β = 0.24, *p* < 0.001), so were paternal and maternal behavioral guidance (father: β = 0.13, *p* = 0.014; mother: β = 0.31, *p* < 0.001), but that of both paternal and maternal harsh discipline were non-significant (father: β = -0.06, *p* = 0.238; mother: β = -0.10, *p* = 0.082). As for the second criterion, mastery goal positively predicted school engagement. For the third criterion, the predictive effects of three interaction terms on school engagement were non-significant.

**FIGURE 3:**
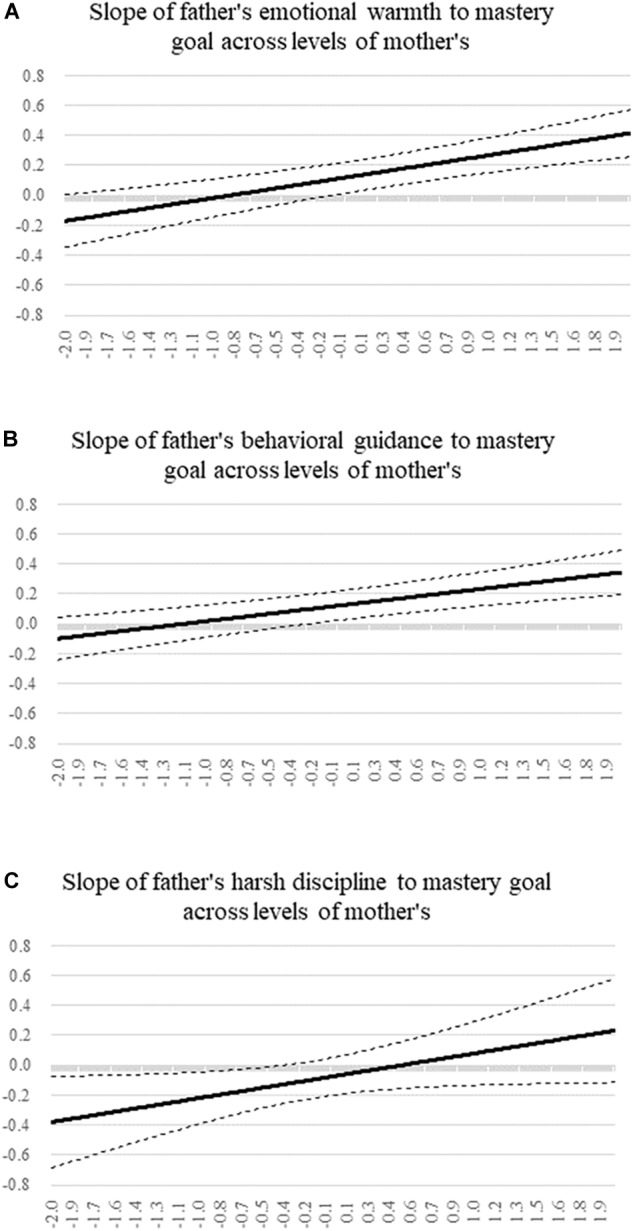
The simple slope of father’s parenting dimensions to mastery goal across levels of mother’s in mediated moderation models. **(A)** The slope of father’s emotional warmth to mastery goal across mother’s scores. **(B)** The slope of father’s behavioral guidance to mastery goal across mother’s scores. **(C)** The slope of father’s harsh discipline to mastery goal across mother’s scores.

The above results suggest that the three mediated moderation models were credible. The moderating effects of maternal parenting behavior dimensions on the relationship between paternal parenting behavior dimensions and school engagement were completely mediated by mastery goal, which supported both H3 and H4. Further, we used the Johnson-Neyman technique to plot changes in the path of each paternal parenting behavior dimension with regard to mastery goal. Both the interaction of paternal and maternal emotional warmth and that of behavioral guidance met the strengthening pattern, while harsh discipline supported the interfering pattern. As Figure 3A,B depicted, the size of the path coefficient from paternal emotional warmth to mastery goal, and from paternal behavioral guidance to mastery goal increased as the maternal corresponding dimension improved. The turning point for the former was -0.10 units, and for the latter was -0.21 units. The two effects were significantly greater than zero when mother scores were equal to or above the points. On the contrary, as shown in Figure 3C, the size of the path coefficient from paternal harsh discipline to mastery goal decreased when maternal harsh discipline increased. The negative effect of paternal harsh discipline on mastery goal was non-significant when mother’s score was over -0.35 units.

### Supplementary Analysis

To determine the extent of the paternal moderating effect, we calculated the effect of the maternal parenting dimension across levels of paternal corresponding dimension using the Johnson-Neyman technique. Again, paternal emotional warmth and behavioral guidance enhanced, while paternal harsh discipline depressed the paths from the maternal corresponding dimensions to school engagement in the simple moderation models, and from the maternal corresponding dimensions to mastery goal in the mediated moderation models. In the simple moderation model, the paths from maternal emotional warmth and behavioral guidance to school engagement were more than zero when the father’s score was equal to or over -1.42 units, and -1.66 units, respectively. The negative effect of maternal harsh discipline was non-significant when the father’s score was over 0.33 units. In the mediated moderation model, with reference to predicting mastery goal, the turning point of the moderating effect was -0.87 units for father’s emotional warmth, -1.67 units for father’s behavioral guidance, and -0.08 units for father’s harsh discipline. Overall, mothers contributed to adolescents’ academic variables across a wider range of scores than fathers.

**FIGURE 4:**
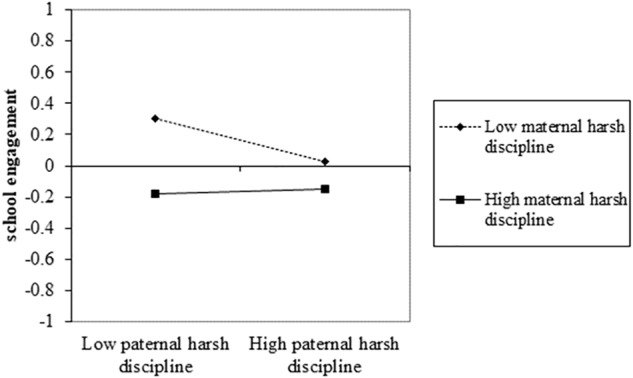
The simple effect of the interaction effect between paternal and maternal harsh discipline on school engagement in the simple moderation model.

**FIGURE 5:**
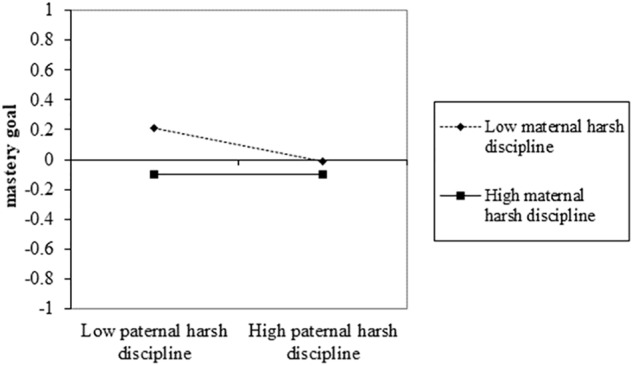
The simple effect of the interaction effect between paternal and maternal harsh discipline on mastery goal in the mediated moderation model.

To more intuitively understand the infrequent interaction effect of paternal and maternal harsh discipline on school engagement in the simple moderation model, and the interaction effect on mastery goal in the mediated moderation model, pick-a-point approach was adopted to describe scores of school engagement and mastery goal when father and mother’s scores of harsh discipline were above or below one standard deviation from the mean. As shown in Figure 4, in the simple moderation model, adolescents’ score of school engagement was only above mean when both parents were low harsh discipline. Once one parent was high harsh discipline, adolescents can experience relatively lower school engagement. Figure 5 showed a similar result of the score of master goal in the mediated moderation model.

## Discussion

Based on family system theory and the self-system model, the present study expanded existing knowledge on the role of parenting behaviors in school engagement among Chinese adolescents. The results indicated that the interaction effects of both parents’ emotional warmth and both parents’ behavioral guidance on school engagement displayed the strengthening pattern, while both parent’ harsh discipline supported the interfering pattern. In addition, the mediated moderation model was supported and all three interactions were mediated by mastery goal. These results underline the importance of viewing family from a systematic perspective and examining the motivational mechanism underlying the relationship between parenting behaviors and academic behavior.

### Parenting Behaviors and School Engagement

The first purpose of this study was to test the overall direct relationship between parental parenting behaviors and school engagement among Chinese adolescents. In line with Hypothesis 1, both fathers and mothers made unique contributions to their offspring’s school engagement, even after controlling for age, gender, and SES.

Parental emotional warmth—parental love, support, and presence with regard to the child—is regarded as supportive parenting behavior in both Western and Chinese cultures (Khaleque, 2013; Yap et al., 2014). Consistent with previous studies, our study provides supportive evidence that both paternal and maternal emotional warmth motivates adolescents to be actively involved in their studies (Bempechat and Shernoff, 2012; Lowe and Dotterer, 2013). Parental warmth provides an emotional foundation for adolescents that enhances their sense of self-efficacy and promotes an internalized sense of competence, which will result in healthy exploration and a higher level of involvement in school activities (Juang and Silbereisen, 2002;Hill and Wang, 2015).

Parental behavioral guidance reflects rational parental teaching and guidance for their children and was developed as an independent dimension in a new parenting tool based on Chinese culture. We considered parental behavioral guidance as a positive control and it was shown to predict school engagement positively in this study. In China, training children is regarded as the responsibility of parents (Chao, 1994; Wu, 1996). Parents provide guidance to help children better understand the purpose of learning, establish good learning habits, and thus promote their willingness to be involved in learning (Patall et al., 2008).

We integrated the content of punishment and psychological control as parental harsh discipline in the new tool, which included both physical punishment and psychological punishment. Parents with a high level of harsh discipline behavior may spank their offspring or threaten to withdraw love if the child fails in school. These negative responses may increase adolescents’ negative affect (e.g., learning-weariness and excessive anxiety) and rebellion, thereby undermine their learning (Grolnick, 2003; Su et al., 2015). In line with this view, our study found that maternal harsh discipline was negatively associated with students’ school engagement (Cheung et al., 2016; Wang et al., 2018). Although the main effect of paternal harsh discipline on students’ school engagement became non-significant when maternal harsh discipline was controlled, the simple effect analysis has shown that it can negatively predict school engagement when maternal harsh discipline was at a low level. More detailed discussion will be presented in the next section.

### The Joint Effect of Father and Mother

According to the family system theory, the effects of the father’s and mother’s parenting behaviors on children’s school engagement are interdependent (Bornstein and Sawyer, 2005). The results of the simple interaction model revealed the joint effect of both parents on adolescents’ school engagement, the effect of one parent’s behavior can be moderated by the other parent’s behavior. Further, our results indicated that different parenting behaviors of the father and the mother follow different interaction patterns.

The interaction effects of both parents’ emotional warmth and behavioral guidance were in accordance with the strengthening pattern. Specifically, mothers’ emotional warmth and behavioral guidance can enhance the positive relationship between the corresponding parenting behavior of fathers and adolescents’ school engagement. One possible explanation is that those supportive mothers may provide a higher level of love, company, and guidance for their children which makes children more open to the influence of the parenting behavior of other important persons such as fathers (Darling and Steinberg, 1993).

However, the interaction effect of paternal and maternal harsh discipline displayed the interfering pattern. The negative relationship between paternal harsh discipline and school engagement was significant when maternal harsh discipline was equal to or below -0.21 units. The negative effect of maternal harsh discipline was significant when the paternal score was equal to or below -0.35 units. It indicates that when one parent’s harsh discipline is high, the negative effect of the other parent’s harsh discipline on school engagement is no longer significant. This interaction pattern is rare but understandable (Foster et al., 2016), as it seems to indicate that the risk of parental harsh discipline exists a ceiling effect. In other words, once one parent is high dominating and controlling, adolescents will develop low levels of school engagement.

In addition, in keeping with previous studies, we also found the dominative effect of mothers on adolescents’ school engagement compared with fathers (Sayer et al., 2004; Martin et al., 2010). Mothers not only scored higher than fathers in all three parenting dimensions, but also contributed more to adolescents’ mastery goal and school engagement. This may be reflective of the fact that mothers spend more time with adolescents (Larson and Richards, 1994; Laible and Carlo, 2004). Although mothers seem to play essential roles in parenting, it does not mean fathers are not important. In fact, adolescents had higher scores on school engagement when both parents scored high in supportive parenting.

### Mediation Effect of Mastery Goal

Our findings also revealed that both paternal and maternal emotional warmth and behavioral guidance indirectly predicted school engagement via mastery goal. The significant mediating effect of mastery goal not only underlines the benefits of mastery goal in improving students’ engagement in learning (Elliot and Church, 1997; Wolters, 2004; Gonida et al., 2009), but also highlights the close link between parental parenting behaviors and mastery goal. Parental emotional warmth and behavioral guidance offer children a sense of emotional security and comfort, make them feel higher self-efficacy, more likely to strive for growth (Trusty and Lampe, 1997) and foster the mastery goal (Duchesne and Ratelle, 2010; Luo et al., 2013), then engaged more in learning (Gonzalez-DeHass et al., 2005; Spera, 2005). By contrast, both parents’ harsh discipline behavior negatively but not significantly predicted mastery goal, which is in line with previous studies (Duchesne and Ratelle, 2010; Fletcher et al., 2012; Chen, 2015; Diaconu-Gherasim and Mãirean, 2016). Controlling parents tend to be more concerned with their children’s grades than skills, they are also inclined to give excessive punishment or praise to encourage their children to excel academically (Gurland and Grolnick, 2005). Consequently, it is possible that adolescents study so as to meet parents’ expectations and to avoid harsh punishment which makes it difficult for adolescents to develop the desire to acquiring knowledge or improve skills based on their own motivation (Dweck, 1986;Duchesne and Ratelle, 2010).

More importantly, all three latent interaction terms significantly predicted school engagement via mastery goal. Similar to the interaction effects on school engagement, both high emotional warmth and behavioral guidance of one parent strengthened the link between the corresponding dimension of the other parent and mastery goal. These findings suggest that adolescents who perceive supportive parenting behavior from both parents are motivated to achieve higher levels of competence than if only one parent possesses high supportive parenting behavior. This, in turn, increases their level of school engagement (Shim et al., 2008; Gonida et al., 2009). However, one parent’s harsh discipline will interfere with the link between the other parent’s harsh discipline and mastery goal. This finding reveals the necessity of examining the interaction effect between paternal and maternal parenting behaviors. Although both paternal and maternal harsh discipline cannot predict mastery goal independently, their interaction effect on mastery goal is found, which indicates that once one parent is high dominating and controlling, adolescents can experience relatively lower motivation to improve their competence, and then lead to lower school engagement.

### Limitations

Limitations of this study cannot be ignored. First, due to the constraints of time and funds, we adopted a cross-sectional design, which inhibits the possibility to explore causal relationships among investigated variables. Also, the idea of “the influential child” (Davidov et al., 2015) was not addressed in this study. According to this idea, the cognitive and behavioral characters of adolescents may, in turn, affect the way parents interact with them. In the future, a longitudinal study can be conducted to understand the dynamic reciprocity between context and learning behaviors.

Second, our results do not adequately explain the effect of one parent’s parenting behavior on the other parent’s parenting behavior. According to research in the field of co-parenting, one parent’s attitude, especially the mother’s, does influence the level of the other parent’s involvement (Yan et al., 2018). To further explore the dynamics of parental parenting behavior, a future study can explore how the parenting behavior of fathers and mothers influence each other.

Finally, all index variables of this study were self-report which may lead to biased results even though large samples were used to reduce the bias. A research setting based on multiple reporting agents will be used in the future.

### Implications for Practice, Application, and Theory

The results of this study have important implications for practice, application, and theory. First, by exploring the interactions between similar parenting behavior of both fathers and mothers, this study found significant joint effects for the parenting behavior of both parents. The interaction patterns of strengthening and interfering seem to indicate that the positive effect of supportive parenting behavior has no upper limit, while the negative effect of non-supportive parenting behavior does. Adolescents can benefit more when both parents are supportive, while their learning motivation and behavior can be affected negatively once one parent is excessive harsh and controlling. This result underlines that both parents are important for parenting, and both of them should try to be more supportive. However, many parents in China always hold different attitudes to their offspring, one plays the villain, and the other plays the hero. This collaborative parenting approach may be not good for the child.

Second, this study showed that behavioral guidance is a parenting behavior that should not be ignored in Chinese culture. Future studies should focus more on the special parenting culture of China.

Finally, mastery goal played a significant mediating role, which supports the importance of children developing competence. To cultivate adolescents’ involvement in learning, it’s necessary for both parenting programs aimed at promoting the usage of supportive parenting behaviors and adolescent programs aimed at guide adolescents to focus more on their self-improvement.

## Conclusion

The present study made a contribution to the family system theory and the self-system model of motivational development. Specifically, paternal and maternal emotional warmth, behavioral guidance can produce both unique and interaction effects on school engagement through motivating adolescents to develop competence. For the interaction effects, one parent’s supportive patenting can intensify the positive role of the other parent’s. However, paternal and maternal harsh discipline can only produce an interaction effect on school engagement via inhibiting the formation of mastery goal. The risk of parental harsh discipline seems to exist a ceiling effect, but it needs to be further tested in future research.

## Data Availability

The raw data supporting the conclusions of this manuscript will be made available by the authors, without undue reservation, to any qualified researcher.

## Ethics Statement

This study was approved by the Ethics Committee of the Faculty of Psychology, Beijing Normal University. Because the potential risk of the protocol was low and the data collection was anonymous, the letter that described the study and consent forms were only sent to school administrators and teachers. Before the data collection, the class adviser sent a message to tell parents about the purpose and voluntary nature of this survey in the Parents WeChat Group. All parents responded in the WeChat Group that they had been informed and agreed to their children’s participation in this survey. Students were also informed of the purpose and voluntary nature of the survey and their right to withdraw at any time.

## Author Contributions

JW contributed to all aspects of work for this study. YY, XS, and HZ contributed to conception and design and revising the manuscript critically. WZ and QX contributed to data.

## Conflict of Interest Statement

The authors declare that the research was conducted in the absence of any commercial or financial relationships that could be construed as a potential conflict of interest.

## References

[B1] Ames C. Archer J. (1988). Achievement goals in the classroom: student learning strategies and motivation processes. *J. Educ. Psychol.* 80 260–267. 10.1037/0022-0663.80.3.260 22091904

[B2] Babinski D. E. Waschbusch D. A. King S. Joyce A. M. Andrade B. F. (2017). Maternal and paternal parenting and associations with school performance in a sample of children with varying levels of externalizing behavior problems. *Sch. Ment. Health* 9 322–333. 10.1007/s10826-006-9066-5

[B3] Barber B. K. (1996). Parental psychological control: revisiting a neglected construct. *Child Dev.* 67 3296–3319. 10.1111/j.1467-8624.1996.tb01915.x 9071782

[B4] Bean R. A. Barber B. K. Crane D. R. (2006). Parental support, behavioral control, and psychological control among African American youth: the relationships to academic grades, delinquency, and depression. *J. Fam. Issues* 27 1335–1355. 10.1177/0192513X06289649

[B5] Bean R. A. Bush K. R. McKenry P. C. Wilson S. M. (2003). The impact of parental support, behavioral control, and psychological control on the academic achievement and self-esteem of African American and European American adolescents. *J. Adolesc. Res.* 18 523–541. 10.1177/0743558403255070

[B6] Bempechat J. Shernoff D. J. (2012). “Parental influences on achievement motivation and student engagement,” in *Handbook of Research on Student Engagement* eds Christenson S. L. Reschly A. L. Wylie C. (New York, NY: Springer) 315–342. 10.1007/978-1-4614-2018-7_15

[B7] Benita M. Roth G. Deci E. L. (2014). When are mastery goals more adaptive? It depends on experiences of autonomy support and autonomy. *J. Educ. Psychol.* 106 258–267. 10.1037/a0034007

[B8] Benner A. D. Graham S. Mistry R. S. (2008). Discerning direct and mediated effects of ecological structures and processes on adolescents’ educational outcomes. *Dev. Psychol.* 44 840–854. 10.1037/0012-1649.44.3.840 18473648

[B9] Boon H. J. (2007). Low- and high-achieving Australian secondary school students: their parenting, motivations and academic achievement. *Aust. Psychol.* 42 212–225. 10.1080/00050060701405584

[B10] Bornstein M. H. Sawyer J. (2005). “Family systems,” in *Blackwell Handbook of Early Childhood Development* eds McCartney K. Phillips D. (Malden, MA: Blackwell) 381–398.

[B11] Bradley R. H. Corwyn R. F. (2002). Socioeconomic status and child development. *Ann. Rev. Psychol.* 53 371–399. 10.1146/annurev.psych.53.100901.135233 11752490

[B12] Bronfenbrenner U. (1986). Ecology of the family as a context for human development: research perspectives. *Dev. Psychol.* 22 723–742. 10.1037/0012-1649.22.6.723

[B13] Castro M. Expósito-Casas E. López-Martín E. Lizasoain L. Navarro-Asencio E. Gaviria J. L. (2015). Parental involvement on student academic achievement: a meta-analysis. *Educ. Res. Rev.* 14 33–46. 10.1016/j.edurev.2015.01.002

[B14] Chao R. K. (1994). Beyond parental control and authoritarian parenting style: understanding Chinese parenting through the cultural notion of training. *Child Dev.* 65 1111–1119. 10.1111/j.1467-8624.1994.tb00806.x 7956468

[B15] Chao R. K. (1995). Chinese and European American cultural models of the self reflected in mothers’ child rearing beliefs. *Ethos* 23 328–354. 10.1525/eth.1995.23.3.02a00030

[B16] Chao R. K. (2001). Extending research on the consequences of parenting style for Chinese Americans and European Americans. *Child Dev.* 72 1832–1843. 10.1111/1467-8624.00381 11768148

[B17] Chao R. Tseng V. (2002). “Parenting of Asians,” in *Handbook of Parenting: Social Conditions and Applied Parenting* Vol. 4 ed. Bornstein M. H. (Mahwah, NJ: Lawrence Erlbaum Associates) 59–93.

[B18] Chen W. W. (2015). The relations between perceived parenting styles and academic achievement in Hong Kong: the mediating role of students’ goal orientations. *Learn. Individ. Differ.* 37 48–54. 10.1016/j.lindif.2014.11.021

[B19] Cheung C. S. Pomerantz E. M. Wang M. Qu Y. (2016). Controlling and autonomy-supportive parenting in the United States and China: beyond children’s reports. *Child Dev.* 87 1992–2007. 10.1111/cdev.12567 27317628

[B20] Darling N. Steinberg L. (1993). Parenting style as context: an integrative model. *Psychol. Bull.* 113 487–496. 10.1037/0033-2909.113.3.487

[B21] Davidov M. Knafo-Noam A. Serbin L. A. Moss E. (2015). The influential child: how children affect their environment and influence their own risk and resilience. *Dev. Psychopathol.* 27 947–951. 10.1017/S0954579415000619 26439055

[B22] Deci E. L. Ryan R. M. (2000). “What is the self in self-directed learning? Findings from recent motivational research,” in *Conceptions of Self-Directed Learning: Theoretical and Conceptual Considerations* ed. Staka G. (Munster: Waxmann) 75–92.

[B23] Diaconu-Gherasim L. R. Mãirean C. (2016). Perception of parenting styles and academic achievement: the mediating role of goal orientations. *Learn. Individ. Differ.* 49 378–385. 10.1016/j.lindif.2016.06.026

[B24] Doctoroff G. L. Arnold D. H. (2017). Doing homework together: the relation between parenting strategies, child engagement, and achievement. *J. Appl. Dev. Psychol.* 48 103–113. 10.1016/j.appdev.2017.01.001

[B25] Duchesne S. Ratelle C. (2010). Parental behaviors and adolescents’ achievement goals at the beginning of middle school: emotional problems as potential mediators. *J. Educ. Psychol.* 102 497–507. 10.1037/a0019320

[B26] Dweck C. S. (1986). Motivational processes affecting learning. *Am. Psychol.* 41 1040–1048. 10.1037/0003-066X.41.10.1040

[B27] Elliot A. J. Church M. A. (1997). A hierarchical model of approach and avoidance achievement motivation. *J. Pers. Soc. Psychol.* 72 218–232. 10.1037/0022-3514.72.1.218 10234849

[B28] Elliot A. J. Thrash T. M. (2002). Approach-avoidance motivation in personality: approach and avoidance temperaments and goals. *J. Pers. Soc. Psychol.* 82 804–818. 10.1037/0022-3514.82.5.804 12003479

[B29] Enders C. K. Bandalos D. L. (2001). The relative performance of full information maximum likelihood estimation for missing data in structural equation models. *Struct. Equ. Modeling* 8 430–457. 10.1207/S15328007SEM0803_5

[B30] Fletcher K. L. Shim S. S. Wang C. (2012). Perfectionistic concerns mediate the relationship between psychologically controlling parenting and achievement goal orientations. *Pers. Individ. Differ.* 52 876–881. 10.1016/j.paid.2012.02.001

[B31] Foster T. D. Froyen L. C. Skibbe L. E. Bowles R. P. Decker K. B. (2016). Fathers’ and mothers’ home learning environments and children’s early academic outcomes. *Read. Writ.* 29 1845–1863. 10.1007/s11145-016-9655-7

[B32] Fredricks J. A. Blumenfeld P. C. Paris A. H. (2004). School engagement: potential of the concept, state of the evidence. *Rev. Educ. Research* 74 59–109. 10.3102/00346543074001059

[B33] Garrett-Peters P. T. Mokrova I. L. Carr R. C. Vernon-Feagans L. Family Life Project Key Investigators (2019). Early student (dis)engagement: contributions of household chaos, parenting, and self-regulatory skills. *Dev. Psychol.* 55 1480–1492. 10.1037/dev0000720 30907606 PMC7017720

[B34] Gershoff E. T. Grogan-Kaylor A. Lansford J. E. Chang L. Zelli A. Deater-Deckard K. (2010). Parent discipline practices in an international sample: associations with child behaviors and moderation by perceived normativeness. *Child Dev.* 81 487–502. 10.1111/j.1467-8624.2009.01409.x 20438455 PMC2888480

[B35] Gonida E. N. Kiosseoglou G. Voulala K. (2007). Perceptions of parent goals and their contribution to student achievement goal orientation and engagement in the classroom: grade-level differences across adolescence. *Eur. J. Psychol. Educ.* 22 23–39. 10.1007/BF03173687

[B36] Gonida E. N. Voulala K. Kiosseoglou G. (2009). Students’ achievement goal orientations and their behavioral and emotional engagement: co-examining the role of perceived school goal structures and parent goals during adolescence. *Learn. Individ. Differ.* 19 53–60. 10.1016/j.lindif.2008.04.002

[B37] Gonzalez-DeHass A. R. Willems P. P. Holbein M. F. D. (2005). Examining the relationship between parental involvement and student motivation. *Educ. Psychol. Rev.* 17 99–123. 10.1007/s10648-005-3949-7 22288600

[B38] Grolnick W. S. (2003). *The Psychology of Parental Control: How Well-Meant Parenting Backfires.* Mahwah, NJ: Erlbaum.

[B39] Gurland S. T. Grolnick W. S. (2005). Perceived threat, controlling parenting, and children’s achievement orientations. *Motiv. Emot.* 29 103–121. 10.1007/s11031-005-7956-2

[B40] Helwig C. C. To S. Wang Q. Liu C. Yang S. (2014). Judgments and reasoning about parental discipline involving induction and psychological control in China and Canada. *Child Dev.* 85 1150–1167. 10.1111/cdev.12183 24936611

[B41] Hill N. E. Wang M. T. (2015). From middle school to college: developing aspirations, promoting engagement, and indirect pathways from parenting to post high school enrollment. *Dev. Psychol.* 51 224–235. 10.1037/a0038367 25485609

[B42] Hoff E. Laursen B. Tardiff T. (2002). “Socioeconomic status and parenting,” in *Handbook of Parenting* 2nd Edn ed. Bornstein M. H. (Mahwah, NJ: Erlbaum) 231–252.

[B43] Janosz M. Archambault I. Morizot J. Pagani L. S. (2008). School engagement trajectories and their differential predictive relations to dropout. *J. Soc. Issues* 64 21–40. 10.1111/j.1540-4560.2008.00546.x

[B44] Jeynes W. H. (2015). A meta-analysis: the relationship between father involvement and student academic achievement. *Urban Educ.* 50 387–423. 10.1177/0042085914525789

[B45] Joussemet M. Landry R. Koestner R. (2008). A self-determination theory perspective on parenting. *Can. Psychol.* 49 194–200. 10.1037/a0012754

[B46] Juang L. P. Silbereisen R. K. (2002). The relationship between adolescent academic capability beliefs, parenting and school grades. *J. Adolesc.* 25 3–18. 10.1006/jado.2001.0445 12009746

[B47] Kaplan A. Maehr M. L. (2007). The contributions and prospects of goal orientation theory. *Educ. Psychol. Rev.* 19 141–184. 10.1007/s10648-006-9012-5

[B48] Khaleque A. (2013). Perceived parental warmth, and children’s psychological adjustment, and personality dispositions: a meta-analysis. *J. Child Fam. Stud.* 22 297–306. 10.1007/s10826-012-9579-z

[B49] Kim S. Y. Wong V. Y. (2002). “Assessing Asian and Asian American parenting: a review of the literature,” in *Asian American Mental Health: Assessment Theories and Methods* eds Kurasaki K. S. Okazaki S. Sue S. (New York, NY: Kluwer Academic) 185–201. 10.1007/978-1-4615-0735-2_13

[B50] Kramer K. Z. (2012). Parental behavioural control and academic achievement: striking the balance between control and involvement. *Res. Educ.* 88 85–98. 10.7227/RIE.88.1.8

[B51] Laible D. J. Carlo G. (2004). The differential relations of maternal and paternal support and control to adolescent social competence, self-worth, and sympathy. *J. Adolesc. Res.* 19 759–782. 10.1177/0743558403260094

[B52] Lam S. F. Jimerson S. Kikas E. Cefai C. Veiga F. H. Nelson B. (2012). Do girls and boys perceive themselves as equally engaged in school? the results of an international study from 12 countries. *J. Sch. Psychol.* 50 77–94. 10.1016/j.jsp.2011.07.004 22386079

[B53] Larson R. Richards M. H. (1994). *Divergent Realities: The Emotional Lives of Mothers, Fathers, and Adolescents.* New York, NY: Basic Books.

[B54] Lau K. L. Lee J. C. (2008). Validation of a Chinese achievement goal orientation questionnaire. *Br. J. Educ. Psychol.* 78 331–353. 10.1111/j.2044-8279.2008.tb00486.x 18039430

[B55] Lawson M. A. Lawson H. A. (2013). New conceptual frameworks for student engagement research, policy, and practice. *Rev. Educ. Res.* 83 432–479. 10.3102/0034654313480891

[B56] Lee J. Yu H. Choi S. (2012). The influences of parental acceptance and parental control on school adjustment and academic achievement for South Korean children: the mediation role of self-regulation. *Asia Pac. Educ. Rev.* 13 227–237. 10.1007/s12564-011-9186-5

[B57] Li X. Meier J. (2017). Father love and mother love: contributions of parental acceptance to children’s psychological adjustment. *J. Fam. Theory Rev.* 9 459–490. 10.1111/jftr.12227

[B58] Li Y. Lerner R. M. (2011). Trajectories of school engagement during adolescence: implications for grades, depression, delinquency, and substance use. *Dev. Psychol.* 47 233–247. 10.1037/a0021307 21244162

[B59] Liem A. D. Lau S. Nie Y. (2008). The role of self-efficacy, task value, and achievement goals in predicting learning strategies, task disengagement, peer relationship, and achievement outcome. *Contemp. Educ. Psychol.* 33 486–512. 10.1016/j.cedpsych.2007.08.001

[B60] Lowe K. Dotterer A. M. (2013). Parental monitoring, parental warmth, and minority youths’ academic outcomes: exploring the integrative model of parenting. *J. Youth Adolesc.* 42 1413–1425. 10.1007/s10964-013-9934-4 23456244

[B61] Luo W. Aye K. M. Hogan D. Kaur B. Chan M. C. Y. (2013). Parenting behaviors and learning of Singapore students: the mediational role of achievement goals. *Motiv. Emot.* 37 274–285. 10.1007/s11031-012-9303-8

[B62] Ma H. Yao M. Ji X. (2015). The influence of parent involvement on students’ school engagement: a mediated moderation model. *Psychol. Dev. Educ.* 31 710–718. 10.16187/j.cnki.issn1001-4918.2015.06.10

[B63] Martin A. Ryan R. M. Brooks-Gunn J. (2010). When fathers’ supportiveness matters most: maternal and paternal parenting and children’s school readiness. *J. Fam. Psychol.* 24 145–155. 10.1037/a0018073 20438190

[B64] Maslowsky J. Jager J. Hemken D. (2015). Estimating and interpreting latent variable interactions: a tutorial for applying the latent moderated structural equations method. *Int. J. Behav. Dev.* 39 87–96. 10.1177/0165025414552301 26478643 PMC4606468

[B65] McKee L. Roland E. Coffelt N. Olson A. L. Forehand R. Massari C. (2007). Harsh discipline and child problem behaviors: the roles of positive parenting and gender. *J. Fam. Violence* 22 187–196. 10.1007/s10896-007-9070-6

[B66] Muller D. Judd C. M. Yzerbyt V. Y. (2005). When moderation is mediated and mediation is moderated. *J. Pers. Soc. Psychol.* 89 852–863. 10.1037/0022-3514.89.6.852 16393020

[B67] Nelson D. A. Hart C. H. Yang C. Olsen J. A. Jin S. (2006). Aversive parenting in China: associations with child physical and relational aggression. *Child Dev.* 77 554–572. 10.1111/j.1467-8624.2006.00890.x 16686788

[B68] Papadaki E. Giovazolias T. (2015). The protective role of father acceptance in the relationship between maternal rejection and bullying: a moderated-mediation model. *J. Child Fam. Stud.* 24 330–340. 10.1007/s10826-013-9839-6

[B69] Patall E. A. Cooper H. Robinson J. C. (2008). Parent involvement in homework: a research synthesis. *Rev. Educ. Res.* 78 1039–1101. 10.3102/0034654308325185

[B70] Pellerone M. Ramaci T. MiccichÉ S. (2018). Identity, Family, relationships among groups and socioeducational disadvantage as factors of school failure: a cross-sectional study in a group of junior high school students of the sicilian Hinterland. *World Futures* 74 321–342. 10.1080/02604027.2018.1492293

[B71] Perris C. Jacobsson L. Linndström H. Knorring L. V. Perris H. (1980). Development of a new inventory for assessing memories of parental rearing behaviour. *Acta Psychiatr. Scand.* 61 265–274. 10.1111/j.1600-0447.1980.tb00581.x 7446184

[B72] Pleck J. H. (2007). Why could father involvement benefit children? Theoretical perspectives. *Appl. Dev. Sci.* 11 196–202. 10.1080/10888690701762068

[B73] Podsakoff P. M. MacKenzie S. B. Lee J. Y. Podsakoff N. P. (2003). Common method biases in behavioral research: a critical review of the literature and recommended remedies. *J. Appl. Psychol.* 88 879–903. 10.1037/0021-9010.88.5.879 14516251

[B74] Pomerantz E. M. Wang Q. (2009). The role of parental control in children’s development in Western and East Asian countries. *Curr. Direct. Psychol. Sci.* 18 285–289. 10.1111/j.1467-8721.2009.01653.x

[B75] Preacher K. J. Rucker D. D. Hayes A. F. (2007). Addressing moderated mediation hypotheses: theory, methods, and prescriptions. *Multivariate Behav. Res.* 42 185–227. 10.1080/00273170701341316 26821081

[B76] Prevatt F. F. (2003). The contribution of parenting practices in a risk and resiliency model of children’s adjustment. *Br. J. Dev. Psychol.* 21 469–480. 10.1348/026151003322535174

[B77] Sangawi H. Adams J. Reissland N. (2015). The effects of parenting styles on behavioral problems in primary school children: a cross-cultural review. *Asian Soc. Sci.* 11 171–186. 10.5539/ass.v11n22p171

[B78] Sarkadi A. Kristiansson R. Oberklaid F. Bremberg S. (2008). Fathers’ involvement and children’s developmental outcomes: a systematic review of longitudinal studies. *Acta Paediatr.* 97 153–158. 10.1111/j.1651-2227.2007.00572.x 18052995

[B79] Sayer L. C. Bianchi S. M. Robinson J. P. (2004). Are parents investing less in children? Trends in mothers’ and fathers’ time with children. *Am. J. Sociol.* 110 1–43. 10.1086/386270

[B80] Schaufeli W. B. Salanova M. González-Romá V. Bakker A. B. (2002). The measurement of engagement and burnout: a two sample confirmatory factor analytic approach. *J. Happiness Stud.* 3 71–92. 10.1023/A:1015630930326

[B81] Shi B. G. Shen J. L. (2007). The relationships among family SES, intelligence, intrinsic motivation and creativity. *Psychol. Dev. Educ.* 23 30–34.

[B82] Shim S. S. Ryan A. M. Anderson C. J. (2008). Achievement goals and achievement during early adolescence: examining time-varying predictor and outcome variables in growth-curve analysis. *J. Educ. Psychol.* 100 655–671. 10.1037/0022-0663.100.3.655

[B83] Skinner E. A. Kindermann T. A. Connell J. P. Wellborn J. G. (2009). “Engagement and disaffection as organizational constructs in the dynamics of motivational development,” in *Handbook of Motivation in School* eds Wentzel K. Wigfield A. (New York, NY: Routledge) 223–245.

[B84] Spera C. (2005). A review of the relationship among parenting practices, parenting styles, and adolescent school achievement. *Educ. Psychol. Rev.* 17 125–146. 10.1007/s10648-005-3950-1 18392544

[B85] Su Y. Doerr H. S. Spinath F. M. Johnson W. Shi J. (2015). The role of parental control in predicting school achievement independent of intelligence. *Learn. Individ. Differ.* 37 203–209. 10.1016/j.lindif.2014.11.023

[B86] Trusty J. Lampe R. E. (1997). Relationship of high-school seniors’ perceptions of parental involvement and control to seniors’ locus of control. *J. Couns. Dev.* 75 375–384. 10.1002/j.1556-6676.1997.tb02353.x

[B87] Van Leeuwen K. (1999). Het meten van opvoeding met de Schaal voor Ouderlijk Gedrag. [Measurement of parenting with the Parental Behavior Scale]. *Diagnostiek-Wijzer* 3 151–170.

[B88] Van Leeuwen K. G. Vermulst A. A. (2004). Some psychometric properties of the ghent parental behavior scale1. *Eur. J. Psychol. Assess.* 20 283–298. 10.1027/1015-5759.20.4.283

[B89] Vasquez A. C. Patall E. A. Fong C. J. Corrigan A. S. Pine L. (2016). Parent autonomy support, academic achievement, and psychosocial functioning: a meta-analysis of research. *Educ. Psychol. Rev.* 28 605–644. 10.1007/s10648-015-9329-z

[B90] Walker C. O. Greene B. A. Mansell R. A. (2006). Identification with academics, intrinsic/extrinsic motivation, and self-efficacy as predictors of cognitive engagement. *Learn. Individ. Differ.* 16 1–12. 10.1016/j.lindif.2005.06.004

[B91] Wang M. Deng X. Du X. (2018). Harsh parenting and academic achievement in Chinese adolescents: potential mediating roles of effortful control and classroom engagement. *J. Sch. Psychol.* 67 16–30. 10.1016/j.jsp.2017.09.002 29571531

[B92] Wang M. Liu L. (2014). Parental harsh discipline in mainland China: prevalence, frequency, and coexistence. *Child Abuse Negl.* 38 1128–1137. 10.1016/j.chiabu.2014.02.016 24661692

[B93] Wang M. Xu W. Wang X. (2017). Parental harsh discipline and adolescents’ academic achievement: mediating of self-disclosure. *Chin. J. Clin. Psychol.* 25 684–690. 10.16128/j.cnki.1005-3611.2017.04.021

[B94] Wang M. T. Eccles J. S. (2012). Adolescent behavioral, emotional, and cognitive engagement trajectories in school and their differential relations to educational success. *J. Res. Adolesc.* 22 31–39. 10.1111/j.1532-7795.2011.00753.x

[B95] Wang M. T. Holcombe R. (2010). Adolescents’ perceptions of school environment, engagement, and academic achievement in middle school. *Am. Educ. Res. J.* 47 633–662. 10.3102/0002831209361209

[B96] Wang Q. Pomerantz E. M. Chen H. (2007). The role of parents’ control in early adolescents’ psychological functioning: a longitudinal investigation in the United States and China. *Child Dev.* 78 1592–1610. 10.1111/j.1467-8624.2007.01085.x 17883450

[B97] Wolters C. A. (2004). Advancing achievement goal theory: using goal structures and goal orientations to predict students’ motivation, cognition, and achievement. *J. Educ. Psychol.* 96 236–250. 10.1037/0022-0663.96.2.236

[B98] Wu D. Y. (1996). “Parental control: psychocultural interpretations of Chinese patterns of socialization,” in *Growing Up the Chinese Way: Chinese Child and Adolescent Development* ed. Lau S. (Hong Kong: Chinese University Press) 1–28.

[B99] Yan J. Schoppe-Sullivan S. J. Kamp Dush C. M. (2018). Maternal coparenting attitudes and toddler adjustment: moderated mediation through father’s positive engagement. *Parenting* 18 67–85. 10.1080/15295192.2018.1444130 31244557 PMC6594390

[B100] Yap M. B. H. Pilkington P. D. Ryan S. M. Jorm A. F. (2014). Parental factors associated with depression and anxiety in young people: a systematic review and meta-analysis. *J. Affect. Disord.* 156 8–23. 10.1016/j.jad.2013.11.007 24308895

